# Impact of COVID-19 on early career scientists: an optimistic guide for the future

**DOI:** 10.1186/s12915-020-00821-4

**Published:** 2020-07-30

**Authors:** Christina M. Termini, David Traver

**Affiliations:** 1 Division of Hematology/Oncology, Department of Medicine, The University of California, Los Angeles, 615 Charles E Young Dr S, Los Angeles, CA 90095 USA; 2 Section of Cell and Developmental Biology, The University of California, San Diego, Natural Sciences Building 6107, La Jolla, CA 92093-0380 USA

Nationally mandated social distancing efforts have been implemented throughout the world during the COVID-19 pandemic to support the health and safety of the public. In response to the pandemic, research institutions have enacted strict changes to permitted research operations, requiring scientists to abide by social distancing guidelines in the laboratory, facility closures, and ramped down laboratory activities. While scientists at all stages in their careers have been impacted by these changes to the research environment, early career scientists such as postdoctoral fellows and junior faculty are particularly vulnerable during these unconventional times. Because early career scientists are in the process of establishing independence during times of restricted research activities, we believe that they are particularly susceptible to the direct and indirect effects of the COVID-19 crisis. While not all researchers will be equally affected by COVID-19 research restrictions, we believe that the impact of such restrictions will be significant and long-lasting. In this piece, we discuss how changes enacted over the past 3 months in response to the COVID-19 pandemic have impacted early career scientists that are members of wet laboratories. We will emphasize how changes in research productivity, the job market, and funding will impact early career scientists in the immediate and late phases of their career. Meanwhile, we will discuss how continued social distancing and limited domestic and international travel will influence early career scientists and, ultimately, the scientific landscape as a whole.

## Research productivity

With a reduction of personnel in laboratories and social distancing measures in place, the traditional laboratory dynamic limits collaborative research that relies on multiple team members working simultaneously. Furthermore, social distancing reduces the natural exchange of ideas that occurs through informal conversations in the laboratory. Depleted of this valuable information sharing channel, junior scientists may not benefit as greatly from the rich training environment offered by a traditional laboratory. To circumvent this loss, we suggest that research advisors sustain scientific creativity and knowledge sharing by holding virtual lab meetings and journal clubs, which can build a sense of community and routine among group members and support research productivity.

Beyond meetings focused on research, we suggest that advisors also hold informal meetings such as social hours to enable scientists to connect in a casual setting, which will further strengthen the team morale that may have been lost during quarantine. Hosting informal meetings can help reduce the stress and anxiety that early career scientists may experience during these uncertain times. We also suggest that early career scientists hold virtual meetings with colleagues and collaborators to update them about their research projects and use this as an opportunity for informal feedback and conceptualization of subsequent studies. As international travel will not be possible in the immediate future, it is necessary for researchers to continue to foster ongoing collaborations using virtual platforms. While virtual interactions may not replace the in-person environment of a laboratory, we are optimistic that digital interactions can still support collaborative research productivity.

During times of social distancing, scientists have reduced access to the laboratory resources needed to move their research forward. While scientists who are capable of working remotely may not experience as strong of an interruption to research productivity, those requiring lab space, core facility support, and specialized equipment may be unable to perform necessary experiments to support their research program. To mitigate this, scientists may consider adapting the direction of their research to take advantage of resources that they do have access to. This may range from seeking training in bioinformatics, computational biology, or molecular modeling to learning how to utilize new analysis software platforms. Some universities are already hosting online courses and workshops on using bioinformatics tools and learning coding languages.

In many cases, it may not be possible to change research direction due to timeline or funding restrictions. We suggest that postdoctoral fellows meet with their research advisors to identify the best mechanisms for maintaining productivity during quarantine and lab distancing, while also taking responsibility for their own productivity. For example, it may be best to use time away from the bench for analyzing data, generating figures, and writing manuscripts. We suggest that postdocs generate specific and achievable goals that they can revisit during subsequent meetings with their advisor. For example, your goals may be to create an outline for a manuscript in preparation, or to generate a specific panel for a figure. By establishing and revising their goals, postdocs can hold themselves accountable for progress and also identify barriers that may exist preventing them from achieving their goals. Finally, time away from the bench can be utilized to apply for funding, from postdoctoral fellowships, career transition awards (NIH K01, K08, K99 mechanisms), or short-term research grants in collaboration with the laboratory head (NIH R03, R21 mechanisms). Acquisition of funding can thus help bridge the gap before fellows apply for independent positions by providing resources to extend fellowship time.

## Timeline

Depending on the nature of the project, time away from the bench will have varying impact on researchers. For example, some scientists may have many of their materials frozen and stored such that they can pick up where they left off when the laboratory re-opens. Meanwhile, in the event that an experiment was terminated prematurely due to research restrictions, the researcher will lose the time that their experiment was incubating in addition to the time spent away from the bench, which can add up to over a year in some cases. Along these lines, it is possible that after re-opening, researchers may have to again ramp down their research programs, leading many to question whether it is appropriate to begin long-term projects. We suggest that researchers think creatively prior to initiating a long-term study to identify concrete stopping points at which a project can be paused or terminated, while still enabling scientists to obtain meaningful data. As such, we believe that the impact of COVID-19 restrictions on the research timeline will be case specific, although all scientists will experience setbacks while working to rebuild their research programs.

Additionally, the restrictions imposed on researchers may extend the publication timeline by preventing submission or resubmission of manuscripts due to incomplete experiments, which may lead to tenure applicants being viewed as less productive than expected. This may have long-term consequences on tenure decisions for a generation of scientists, and we may see increased departure of the academic workforce if they are held to pre-pandemic standards. To circumvent this change in productivity, many departments have provided junior faculty with an extension in their tenure application timelines.

For postdoctoral fellows, first-author publications are a crucial metric for future employment. If fellows are unable to publish as planned, they may be perceived as unproductive by future employers and, thus, a generation of untapped talent be excluded from research landscape. One solution to this problem is to extend the postdoctoral timeline, but contract extension may not be possible for scientists with funding restrictions or for international scientists on strict VISA timelines. We suggest that research advisors meet with their fellows to discuss potential strategies to support alternative employment possibilities, such as contract extension or in some cases, promotion to project scientist or research/adjunct professor. Additionally, advisors may consider supporting non-traditional publication mechanisms, such as Registered Reports, depositing the manuscript on a preprint server to bring the study to the attention of the community before publication in a journal, or adapting a larger story to a brief reports format. Journals are critical intermediaries in the publication process, and some of them have already adopted measures to support the publication process, such as extending the submission deadlines and working with researchers on revision plans to take into account the challenges of this restricted time. For example, journals of Springer Nature, Elsevier, Cell Press, EMBO Press, eLife, and many others have publicized practices to support the dissemination of research findings during the COVID-19 crisis. Keeping the lines of communication open is essential, and we would prompt researchers to be proactive in their discussions with the editorial staff about these challenges.

## Funding

Along these same lines, scientific productivity is used as a metric for funding decisions across the world. Given the impact of COVID-19 restrictions on research productivity, it is unlikely that the rising generation of scientists will be able to compete for funding resources if scientists are to be held to the pre-pandemic standards. Without sufficient funding, early-stage investigators may be unable to continue their research programs as planned, thereby preventing their scholarly contributions from being realized. We suggest that grant reviewers consider how changes induced by COVID-19 may have impacted researchers. More specifically, funding agencies may consider allowing scientists to write an addendum to their application to describe how their research program and institution were impacted by COVID-19. This may provide researchers with an opportunity to explain that timepoints were omitted from an analysis because they did not have access to the laboratory, or that experiments were performed with an alternative animal strain because researchers were unable to order animals from outside vendors. This would give researchers an opportunity to acknowledge the shortcomings of studies while explaining that certain unconventional choices were made during a time of restricted resources.

## Networking

Networking represents a crucial opportunity to support the career development of scientists at all stages in their career and is particularly important for early-stage scientists working to build their scientific community. A majority of networking interactions occur via in-person interactions at conferences, seminars, and other events whether through formal meetings or informal introductions. To comply with social distancing restrictions, many societies have postponed or canceled their scientific events, significantly changing the mechanisms by which networking can occur [1]. We suggest that researchers can still take advantage of scientific networking at virtual meetings by reaching out to scientists in the audience through follow-up emails. Social media involvement using Twitter and LinkedIn also represent powerful mechanisms to promote networking with the international scientific community. Additionally, several Slack channels have been generated that allow researchers with common interests to engage in discussions at their own liberty, which will help scientists generate new connections with researchers around the world.

With reduced in-person interactions, early career scientists also have limited opportunities to promote their science and research vision, which may prevent them from reaching larger audiences to disseminate their results. In fact, however, it seems that virtual seminars or conferences may actually reach a larger audience because scientists do not have to travel to attend and the conference fees are sometimes reduced. However, we do not yet understand how presenting our work in the digital realm may impact situations in which keeping results confidential or without sharing is preferred. As early-stage scientists are at particularly vulnerable stages in our careers, scientists should carefully consider what information to share publicly as participants may have digitized access to sensitive information or novel ideas. However, participation in virtual presentations can also be incredibly beneficial by facilitating collaborations that may not have been forged previously.

## Job market

The job market has been significantly modified by the COVID-19 pandemic, with many open searches being put on hold indefinitely, leaving a generation of fellows unable to transition to the next stage in their career. For postdocs nearing the end of their contracts, it may be necessary to seek employment in non-academic sectors before entering the job market or face unemployment. Working outside of academia may equip researchers with new perspectives and expertise, and we are hopeful that employers and funding agencies will recognize this and not penalize fellows looking to re-enter academia. Furthermore, in the event that job searches are continuing, most interviews are taking place using virtual platforms, which can be an unfamiliar experience for interviewees to navigate. We strongly suggest that postdoctoral fellows utilize time away from the bench to practice key job skills such as interviewing and presentation skills for seminars and chalk talks using digital platforms such as Zoom. By using this time to refine these skillsets, applicants will be better prepared for the interview process when the time comes.

Additionally, we hope that hiring committees will take into consideration the impact of the pandemic on the publication record of applicants from specific fields to ensure representation is given to fields that have been more drastically affected by laboratory limitations. Again, a solution to this problem may be to generate a short narrative to include in the application packet to how restrictions incurred from the COVID-19 pandemic specifically impacted the applicant to ensure that a diverse group of scientists remains in the scientific community. For recently hired junior faculty, university-mandated hiring freezes will likely limit the ability to bring in necessary staff and trainees to support the research vision of your laboratory. However, it may be necessary to communicate your staffing needs to your Department Chair, who may advocate to decision-makers to enable hiring, which will ultimately support the research of junior faculty.

## Conclusions and outlook

The scientific community as a whole will be even more drastically impacted by the COVID-19 pandemic if early career scientists are not supported. Early career researchers represent the next generation of leaders in scientific research, bringing with them unique expertise, creativity, and ideas for the future of biological research. To ensure that a generation of scientists is not lost, we encourage advisors, colleagues, and collaborators to offer help and support. We also encourage early career researchers to lean on their community for support and guidance. Whether it be through virtual mentoring sessions, online seminars, or informal meetings between scholars, we believe that discussing our unique experiences and concerns for the future will help scientists persevere and grow during and following the pandemic.
